# Direct and sustained intracellular delivery of exogenous molecules using acoustic-transfection with high frequency ultrasound

**DOI:** 10.1038/srep20477

**Published:** 2016-02-04

**Authors:** Sangpil Yoon, Min Gon Kim, Chi Tat Chiu, Jae Youn Hwang, Hyung Ham Kim, Yingxiao Wang, K. Kirk Shung

**Affiliations:** 1 Department of Biomedical Engineering, University of Southern California, Los Angeles, California 90089, USA; 2 Department of Information and Communication Engineering, Daegu Gyeongbuk Institue of Science & Technology, Daegu, Korea; 3 Department of Bioengineering & Institute of Engineering in Medicine, University of California, San Diego, La Jolla, California 92093, USA; 4 Analogic Corporation, 8 Centennial Drive, Peabody, MA 01960, USA

## Abstract

Controlling cell functions for research and therapeutic purposes may open new strategies for the treatment of many diseases. An efficient and safe introduction of membrane impermeable molecules into target cells will provide versatile means to modulate cell fate. We introduce a new transfection technique that utilizes high frequency ultrasound without any contrast agents such as microbubbles, bringing a single-cell level targeting and size-dependent intracellular delivery of macromolecules. The transfection apparatus consists of an ultrasonic transducer with the center frequency of over 150 MHz and an epi-fluorescence microscope, entitled acoustic-transfection system. Acoustic pulses, emitted from an ultrasonic transducer, perturb the lipid bilayer of the cell membrane of a targeted single-cell to induce intracellular delivery of exogenous molecules. Simultaneous live cell imaging using HeLa cells to investigate the intracellular concentration of Ca^2+^ and propidium iodide (PI) and the delivery of 3 kDa dextran labeled with Alexa 488 were demonstrated. Cytosolic delivery of 3 kDa dextran induced via acoustic-transfection was manifested by diffused fluorescence throughout whole cells. Short-term (6 hr) cell viability test and long-term (40 hr) cell tracking confirmed that the proposed approach has low cell cytotoxicity.

Transfection of macromolecules to the cytoplasm of cells remains a challenging problem because the lipid bilayer of the cell membrane acts as a barrier to foreign molecules. However, the intracellular delivery of membrane-impermeable molecules with high efficiency and minimum side effects is a crucial process for laboratory and clinical applications^1^. For example, reprogramming of stem cells using protein^2^, cell labeling and tracking of single molecules using quantum dots^3^,^4^ and the visualization of cell-to-cell interaction using fluorescence resonance energy transfer (FRET)-based biosensors^5^,^6^ have been interesting applications in cell biology. Therefore, a variety of methods for intracellular delivery of macromolecules into the cytoplasm or nucleus has been developed using lipids and polymers^7^,^8^, viral vectors^9^,^10^, electroporation^11^,^12^, photo-transfection^13^,^14^, shear permeabilization^15^, microfluidic channel with constrictions^16^, and microinjection^3^,^17^,^18^,^19^,^20^. Although transfection with lipids, polymers, and viral vectors is usually efficient, deliverable molecules are limited to DNA and RNA. Furthermore, cargoes usually cannot be targeted to specific individual cells as desired. Electroporation has a high level of cytotoxicity and it can aggregate small molecules such as quantum dots and nanoparticles. Photo-transfection is also utilized to deliver target molecules into a cell. However, photo-toxicity may cause damage to cells, particularly when shorter wavelengths of light are used. Microinjection is the most direct method, which allows the injection of almost all kinds of molecules into any kind of cells; however, this technique has relatively low throughput compared to other methods. The shear permeabilization method can also be utilized for delivery but has lower cell viability than microinjection.

Low frequency ultrasound and microbubble-based cell membrane disruption, called sonoporation, has been investigated^21^,^22^,^23^,^24^,^25^. With low frequency ultrasound, approximately between 1 and 5 MHz, the affected number of cells by the generated ultrasound field is usually quite large because the focal area of low frequency ultrasound is in the millimeter range. To specifically target and concentrate ultrasound energy to cells for higher transfection efficiency, microbubbles, which are conjugated with functional moiety, are attached to target cells or free microbubbles are suspended in the mixture of solution with cells. Microbubble dynamics are very complicated and should be investigated intensively to better understand the mechanism of sonoporation^26^,^27^. Microbubble dynamics during low and high intensity insonation behave quite differently. The high intensity ultrasound field induces an abrupt collapse and the formation of microjets with shock wave propagation, which may cause damage to cells. However, low intensity ultrasound field results in much weaker and stable bubble oscillation, which has different effects on single cells^27^.

Calcium is a versatile molecule that predicts many cell phenotypes such as cell differentiation and death^28^. Therefore, monitoring intracellular concentration of Ca^2+^ provided direct readout of the cell viability after the application of acoustic pulses^29^. A fluorescence resonance energy transfer (FRET)-based Ca^2+^ biosensor is an exquisite tool to visualize molecular activities with subcellular targeting capability, which is much less invasive than microinjection and patch clamping^30^,^31^. FRET-based Ca^2+^ biosensor uses Calmodulin (CaM) and M13 as an interacting pair (Supplementary Figure 3)^5^,^32^,^33^ to provide dynamic readouts of intracellular Ca^2+^ concentration in live cells. Binding of Ca^2+^ between CaM and M13 results in the FRET ratio increase, which indicates the Ca^2+^ influx into cell cytoplasm (Supplementary Figure 3). This genetically encoded molecular biosensor can target subcellular regions to visualize more accurate Ca^2+^ concentration than fluorescence dye.

In this paper, acoustic-transfection using high frequency ultrasonic pulses was introduced as a new technique to remotely perturb the lipid bilayer of the cell membrane and to deliver exogenous molecules into a targeted single-cell. Acoustic-transfection is designed to deliver macromolecules with various sizes and structures into designated cells due to single-cell targeting capability. One advantage of single-cell level transfection over bulk transfection is that two neighboring single cells can express different cargo (or biosensor) proteins and allow the simultaneous monitoring of different molecular events in these neighboring cells. Each ultrasonic pulse was focused into a small confined area, similar in size to a single cell, to achieve single-cell level targeting without microbubbles by an ultrasonic transducer with a center frequency over 150 MHz. The beam width of an acoustic pulse was 10 μm or less and the position of the acoustic pulse was pinpointed at desired locations within a targeted single-cell with automated 3D stages. By adjusting input parameters of acoustic pulse, desired molecules for the intracellular delivery can be chosen depending on their size. After an acoustic pulse was applied to a targeted single-cell to induce the intracellular delivery of molecules, i.e., acoustic-transfection, the cell was monitored by an epi-fluorescence microscope^34^,^35^,^36^. A live cell imaging with HeLa cells using FRET-based Ca^2+^ biosensor and propidium iodide (PI) was performed simultaneously to explore the relationship between the cell viability and acoustic pulses and to demonstrate the amount of delivered molecules inside a targeted single-cell depending on the size of molecules and the input parameters of acoustic pulses. Input parameters controlled the strength and frequency of acoustic pulses. Because living cells maintain their intracellular calcium concentration in their cytosol, Ca^2+^ induced FRET fluorescence signal tends to comes back to original level quickly. PI emits fluorescence signal only when it binds to nucleic acids. PI is retained in the nucleus region of cells for a long time. When an acoustic pulse induces cell death with impaired cell plasma membrane, PI signal can maintain a high level of signals for a sustained period because of the continuous influx of PI molecules. However, FRET signal may decrease because of the efflux of FRET biosensor. The intracellular delivery of macromolecules, i.e., 3 kDa dextran labeled with Alexa 488, was also investigated with two different input parameters that did not induce cell death. Short-term (6 hr) cell viability test and long-term (40 hr) cell tracking were performed to assess the effect of acoustic pulses on a targeted single-cell. Direct and sustained intracellular delivery of macromolecules, which utilizes a high frequency ultrasound-induced perturbation on the cell membrane, was introduced and single-cell level targeting capability and low cytotoxicity of acoustic-transfection was established.

## Results

### Acoustic-transfection system and input parameters

**Figure 1:**
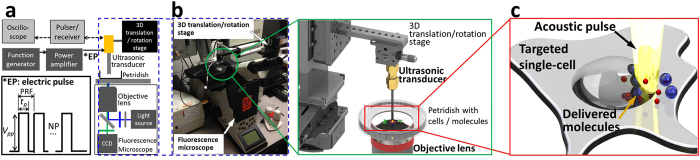
Acoustic-transfection system. (**a**) A schematic view of the experimental system. The position of an ultrasonic transducer is controlled by 3D translation/rotation stage. The focus of the ultrasonic transducer is located on the surface of a petridish using a pulser/receiver and an oscilloscope (bright gray box) by finding a maximum echo time response (Supplementary Figure 1). Electric pulse (EP) is generated by a function generator and a power amplifier (dark gray box). Four input parameters for EP are peak-to-peak voltage (*Vpp*), pulse duration (*tp*), pulse repetition frequency (PRF), and number of pulses (NP). (**b**) Picture of acoustic-transfection system and detailed view of a petridish with cells and molecules. The foci of an objective lens and an ultrasonic transducer are aligned. (**c**) An acoustic pulse is generated (yellow beam), after EP triggers an ultrasonic transducer. The plasma membrane of a targeted single-cell is perturbed by the acoustic pulse and the intracellular delivery of molecules is induced through the perturbed area.

We have developed acoustic-transfection systems to induce and monitor the intracellular delivery of macromolecules as shown in Fig. 1a (See Materials and Methods for details). We hypothesize that many exogenous molecules can be delivered into a targeted single-cell through the perturbed area on cell membrane as shown in Fig. 1c. The amount and the size of delivered molecules by acoustic-transfection have been found to depend on the strength and the duration of each acoustic pulse and the frequency and the number of acoustic pulses. These four factors were determined by input parameters of electric pulse (EP) such as peak-to-peak voltage (*V*_*pp*_), pulse duration (*t*_*p*_), pulse repetition frequency (PRF), and the number of electric pulse (NP) as shown in Fig. 1a.

**Figure 2:**
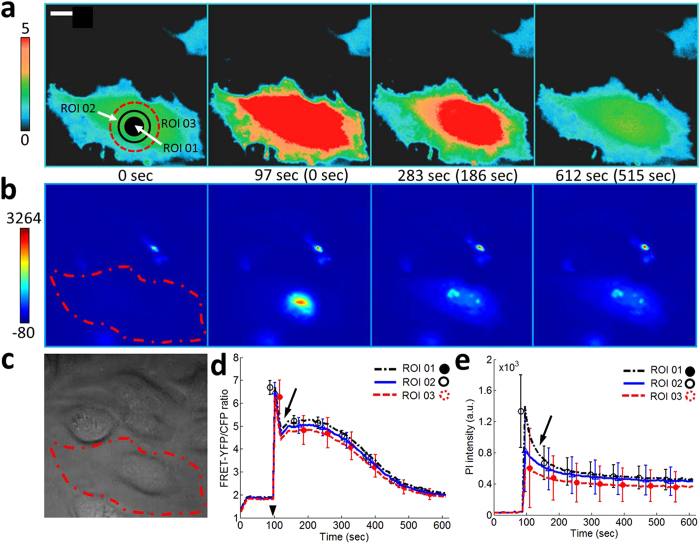
Upper limit of input parameters not to induce cell death with *V*_*pp*_ of 22 V. (**a**) Color images and (**d**) the time courses of the FRET-YFP/CFP ratio of Ca^2+^ biosensor at region of interest (ROI) 01, 02, and 03 represent extremely rapid increase of the intracellular Ca^2+^ concentration right after the application of an acoustic pulse with input parameters of *t*_*p*_ = 30 μs, PRF = 0, and NP = 1. A rebound of FRET-YFP/CFP ratio after the solid arrow at (**d**) indicates that Ca^2+^ influx continues. The color scale bar on the left at (**a**) represents the range of emission ratio with black and red colors, indicating low and high levels of Ca^2+^ concentration, respectively. (**b**) Color images and (**e**) the time courses of PI intensity at the same ROIs, obtained by a simultaneous live cell imaging, show that the PI molecules spreads throughout the whole cell after the initial and strong influx of PI molecules through ROI 01. The color scale bar indicates the range of PI intensity with blue (low) and red (high) levels of PI concentration. The image in (**c**) shows DIC image. The scale bar represents 10 μm. The error bars represent plus and minus one standard deviation. The parenthesis between subfigures A and B indicates the time after the onset of acoustic-transfection. Arrow head in (**d**) indicates the application of acoustic pulse.

**Figure 3:**
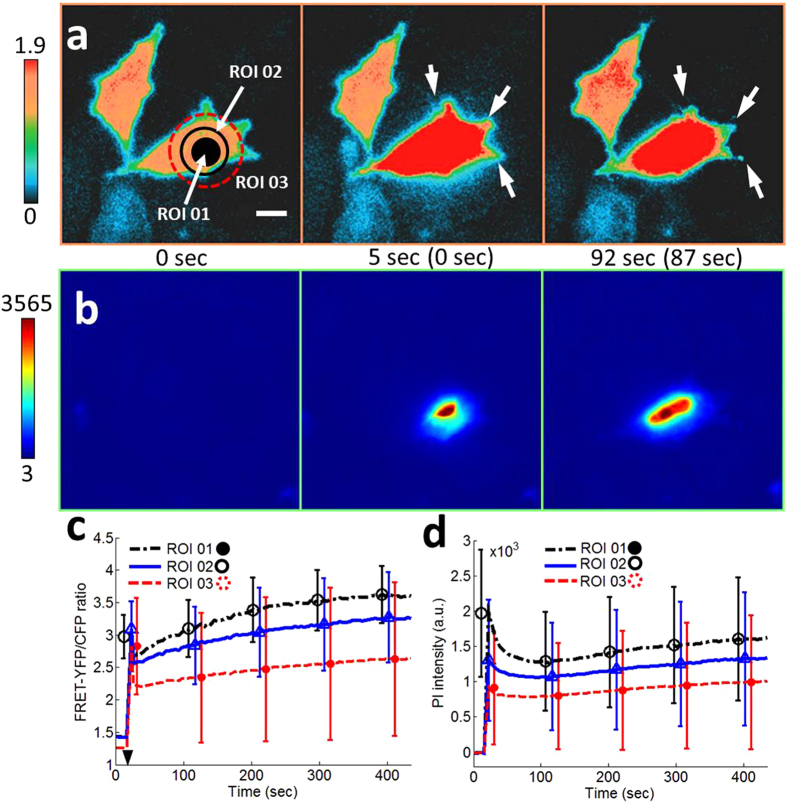
Cell death observation by strong acoustic pulse with *V*_*pp*_ = 22V. (**a**) Color images and (**c**) the time courses of the FRET-YFP/CFP ratio of Ca^2+^ biosensor at region of interest (ROI) 01, 02, and 03 represent cell death. White arrows indicate cell blebbing. The input parameter of an acoustic pulse was (*t*_*p*_ = 35 μs, PRF = 0, and NP = 1). The color scale bar on the left at (**a**) represents the range of emission ratio with black and red colors, indicating low and high levels of Ca^2+^ concentration, respectively. (**b**) Color images and (**d**) the time courses of PI intensity at the same ROIs, obtained by a simultaneous live cell imaging, show extremely strong influx of PI molecules. The color scale bar indicates the range of PI intensity with blue (low) and red (high) levels of PI concentration. Both FRET-YFP/CFP ratio and PI intensity increases, which is the evidence of cell death and irreversible cell membrane disruption. Arrow head in (**c**) indicates the application of acoustic pulse. In Supplementary Figure 6, FRET-YFP and CFP channel data are presented. The parenthesis between subfigures A and B indicates the time after the onset of acoustic-transfection. The scale bar represents 10 μm.

First, we wanted to find input parameters to induce the death of a targeted single-cell. We found that conditions I (*V*_*pp*_ = 22 V, *t*_*p*_ = 30 μs, PRF = 0, and NP = 1) and II (*V*_*pp*_ = 43V, *t*_*p*_ = 10 μs, PRF = 0, and NP = 1) were two possible upper limits of input parameters right before inducing cell death. Simultaneous live cell fluorescence imaging using FRET-based Ca^2+^ biosensor and propidium iodide (PI) indicates that conditions I and II induce reversible cell membrane disruption as shown in Fig. 2 and Supplementary Figure 4. Under conditions I and II, FRET ratio (Fig. 2a,d and Supplementary Figure 4) eventually comes back to normal level and the fluorescence intensity of PI (Fig. 2b,e and Supplementary Figure 4) slowly decreases as time advances. FRET ratio and PI intensity at three regions of interest (ROI) within targeted single-cells are plotted in Fig. 2d,e and Supplementary Figure 4 for conditions I and II, respectively. ROI 01 corresponds to the focal area of an ultrasonic transducer, so the initial perturbation on the cell membrane starts at ROI 01. The spikes of PI intensity at ROI 01 in Fig. 2e and Supplementary Figure 4 indicate that the initial and strong influx of PI occurs through ROI 01. Trapped PI molecules inside a targeted single-cell spread out to mainly nucleus area of the cell. For conditions I and II, FRET ratio plots show rebounds, indicated by solid arrows in Fig. 2d and Supplementary Figure 4, while PI intensity plots show continuous intensity decrease. These results suggest that the perturbed area near ROI 01 is initially larger than both Ca^2+^ and PI molecules but the perturbed area may have shrunk soon, precluding PI molecules from entering into the targeted single-cell. Slight increase of *t*_*p*_ from 30 μs to 35 μs for condition I and from 10 μs to 12 μs for condition II induced cell death (Fig. 3, Supplementary Figure 5 and Supplementary Video 1). Under the new input parameters with longer *t*_*p*_, targeted single-cells were dead and obvious cell blebbing was detected (white arrows in Fig. 3 and Supplementary Figure 5). PI intensity kept increasing, indicating a permanent cell membrane disruption as shown in Fig. 3 and Supplementary Figure 5. Arrow heads in Figs 2d and 3c indicate the moment when an acoustic pulse was applied. Both FRET signals and PI intensities changed within one second in response to an acoustic pulse.

### Size dependent intracellular delivery

**Figure 4:**
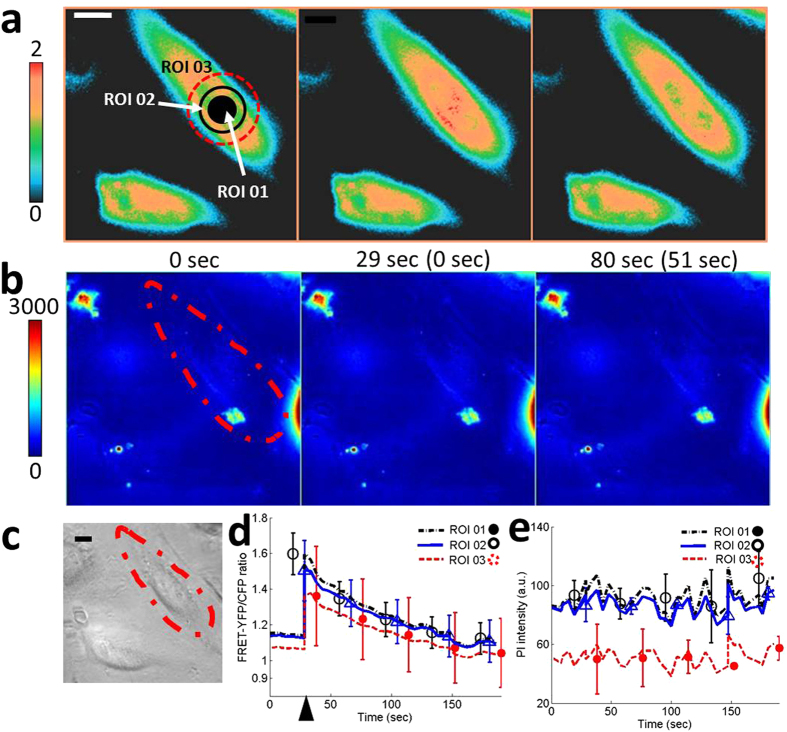
Controlled intracellular delivery with different molecular sizes. (**a**) Color images and (**d**) the time courses of the FRET-YFP/CFP ratio of Ca^2+^ biosensor at region of interest (ROI) 01, 02, and 03 represent increase of the intracellular Ca^2+^ concentration right after the application of an acoustic pulse with input parameters of *V*_*pp*_ = 47 V, *t*_*p*_ = 0.875 μs, PRF = 0, and NP = 1. FRET-YFP/CFP ratio shows a small increase, indicating a small influx of Ca^2+^ into a targeted single-cell. The color scale bar on the left at (**a**) represents the range of emission ratio with black and red colors, indicating low and high levels of Ca^2+^ concentration, respectively. (**b**) Color images and (**e**) the time courses of PI intensity at the same ROIs, obtained by a simultaneous live cell imaging, show no influx of PI molecules. The image in (**c**) show DIC image. The scale bar represents 10 μm. The parenthesis between subfigures A and B indicates the time after the onset of acoustic-transfection. Arrow head in (**d**) indicates the application of acoustic pulse.

Next, an experiment is carried out to determine the effect of the size of molecules on the intracellular delivery. Because Ca^2+^ is smaller than propidium iodide (PI) molecules^37^, only Ca^2+^ delivery is induced if appropriate input parameters are chosen. We decrease pulse duration (*t*_*p*_) until we only see FRET ratio changes while PI intensity stays the same. One acoustic pulse (PRF = 0 and NP = 1) with a peak-to-peak voltage (*V*_*pp*_) of 47 V and a *t*_*p*_ of 0.875 μs induces changes only in FRET ratio as shown in Fig. 4d,e. Comparing FRET-YFP/CFP ratio plot in Fig. 4d to plots in Fig. 2d and Supplementary Figure 4D, FRET ratio is observed to decrease monotonically after the initial jump at 29 sec without having a rebound. Arrow head in Fig. 4d indicates the moment when an acoustic pulse was applied. FRET signals changed within one second in response to an acoustic pulse.

### Accumulation of molecules from repeated intracellular delivery

**Figure 5:**
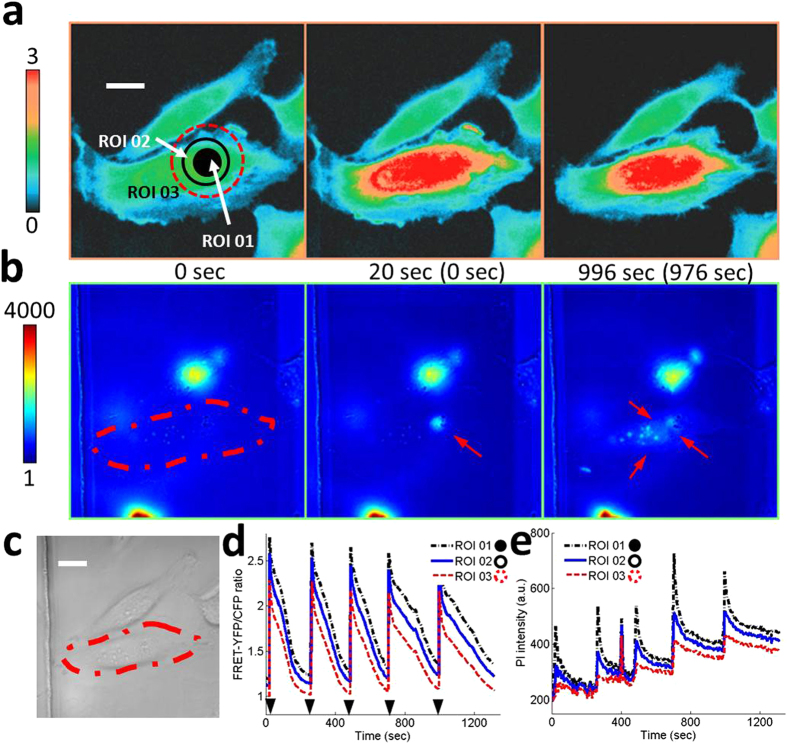
Accumulation of molecules by repeated intracellular delivery. Five repeated acoustic pulses with *V*_*pp*_ = 47 V and *t*_*p*_ = 2 μs are applied at ROI 01. (**a**) Color images and (**d**) the time courses of the FRET-YFP/CFP ratio of Ca^2+^ biosensor at ROIs 01, 02, and 03 represent repeated increase and decrease of the intracellular Ca^2+^ concentration. The color scale bar on the left represents the range of FRET ratio with black and red colors, indicating low and high levels of Ca^2+^ concentration, respectively. (**b**) Color images and (**e**) the time courses of PI intensity at the same ROIs show that the PI molecules gradually accumulate inside a target cell, indicated by solid arrows at the second and third panels in (**b**). The color scale bar indicates the range of PI intensity with blue (low) and red (high) levels of PI concentration. The image in (**c**) show DIC image. The scale bar represents 10 μm. The parenthesis between subfigures A and B indicates the time after the onset of acoustic-transfection. Arrow heads in (**d**) indicate the application of acoustic pulse.

To introduce desired amount of molecules into targeted single-cells, multiple applications of an acoustic pulse were investigated. Each acoustic pulse should be as gentle as possible to generate moderate perturbations on the cell membrane and to keep a targeted single-cell in good condition. Five consecutive acoustic pulses at 20, 265, 487, 706, and 996 second were applied to the targeted single-cell with *V*_*pp*_ of 47 V and *t*_*p*_ of 2 μs. FRET ratio plot indicates that Ca^2+^ level in the cytoplasm of the targeted single-cell returns to normal in approximately 4 to 5 minutes after the application of each acoustic pulse as shown in Fig. 5d and Supplementary Video 2. No rebound in FRET ratio is observed. PI intensity increases stepwise after each acoustic pulse (Fig. 5e and Supplementary Video 3). After five acoustic pulses, PI intensity increases from 200 to 400 as shown in Fig. 5e. Fluorescence images at the second (*t* = 20 sec) and the third (*t* = 996 sec) panels in Fig. 5b show the direct evidence of PI accumulation in nucleus region as indicated by solid arrows. Arrow heads in Fig. 5d indicate the moments when acoustic pulses were applied. Both FRET signals and PI intensities changed within one second in response to an acoustic pulse.

### Intracellular delivery of macromolecules and 40 hours of cell tracking

**Figure 6:**
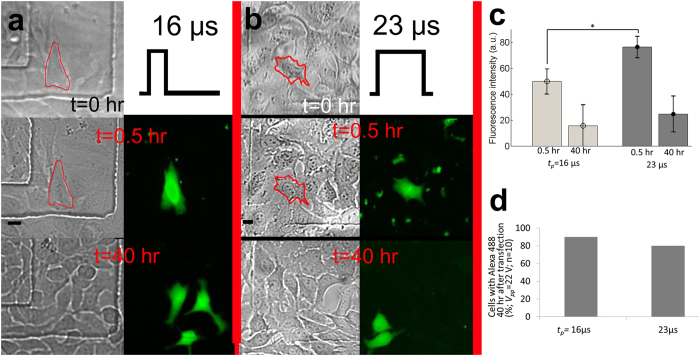
Intracellular delivery of 3 kDa dextran. Intracellular delivery of 3 kDa dextran labeled with Alexa 488 using two different pulse durations (*t*_*p*_) of (**a**) 16 μs (n = 10) and (**b**) 23 μs (n = 10). The second and the third rows in A and B represent bright-field and fluorescence images of treated cells after 30 minutes and 40 hours after the treatment, respectively. Scale bars indicate 20 μm. Diffused green fluorescence from treated cells is observed, which indicates cytosolic staining of 3 kDa dextran labeled with Alexa 488. (**c**) Fluorescence intensity measurements of targeted single-cells 0.5 hr and 40 hr after the treatment. Asterisk (*) indicates statistically significant differences between the amount of delivered 3 kDa dextran into target cells, treated with different *t*_*p*_. Error bars indicate plus/minus one standard deviation. (**d**) 40 hours of cell tracking results after the acoustic-transfection. 90% and 80% of treated cells were alive and divided into daughter cells when *t*_*p*_ was 16 μs and 22 μs, respectively (Supplementary Figures 7 and 8 for details).

Figure 6a,b represent the successful intracellular delivery of 3 kDa dextran labeled with Alexa 488 into targeted single-cells using one acoustic pulse (PRF = 0 and NP = 1) with the pulse durations (*t*_*p*_) of 16 μs and 23 μs, respectively. The peak-to-peak voltage (*V*_*pp*_) was 22 V. The input parameters are in safe range according to a live cell imaging using FRET biosensor and PI. However, to double check the effects of acoustic pulses on targeted single-cells, short-term (6 hr) cell viability test was also conducted. 100% cells are viable (Supplementary Figure 9). Solid lines in bright-field images at the first and the second rows of Fig. 6a,b indicate targeted single-cells before and 0.5 hr after the treatment. Bright-field and fluorescence images of targeted single-cells 0.5 hr and 40 hours after the application of an acoustic pulse are shown in the second and the third rows in Fig. 6a,b, respectively. After 40 hours of incubation (the third row in Fig. 6a,b), daughter cells emit fluorescence signal in the same wavelength region as originally targeted cells, indicating that the delivered 3 kDa dextran molecules were transferred to daughter cells from the originally targeted single-cells (the second row in Fig. 6a,b). We measured fluorescence intensity of targeted single-cells 0.5 hr and 40 hr after acoustic-transfection as shown in Fig. 6c. Mean values and standard deviations are 49.76 ± 9.5 for *t*_*p*_ = 16 μs (n = 8) and 76.38 ± 7.9 for *t*_*p*_ = 23 μs (n = 7), measured 0.5 hr after acoustic-transfection and measured intensities are statistically significantly different, which indicates the difference between amounts of delivered dextran molecules under two different *t*_*p*_ (Asterisk (*) in Fig. 6c, *p*-value = 0.006). However, after 40 hr, fluorescence intensities of targeted single-cells are not significantly different with the mean values and standard deviations of 15.60 ± 15.0 for *t*_*p*_ = 16 μs and 24.57 ± 13.6 for *t*_*p*_ = 23 μs. This may come from the degradation of Alexa 488. Fluorescence intensity measurement long after the acoustic-transfection does not have significant implications. We counted number of cases that had daughter cells with the same fluorescence wavelength as Alexa 488 after 40 hours of incubation (n = 10). Figure 6d presents 40 hour cell tracking results. A 90% and 80% of targeted single-cells were alive and divided into daughter cells 40 hours after the acoustic transfection with *t*_*p*_ of 16 μs and 23 μs, respectively (Supplementary Figure 7 and 8).

## Discussion

In this paper, an approach for intracellular delivery of macromolecules using high frequency ultrasound was successfully demonstrated. The proposed approach has a single-cell selectivity and low cytotoxicity. Only targeted cells from a monolayer of HeLa cells experienced the increase in PI intensity and emitted fluorescence from 3 kDa dextran labeled with Alexa 488 (Fig. 6 and Supplementary Figures 7 and 8). Short-term (6 hr) cell viability test using a LIVE/DEAD Cell Imaging kit (Supplementary Figure 9) and long-term (40 hr) cell tracking (Supplementary Figures 7 and 8) confirmed that the proposed approach had low cell cytotoxicity. Treated cells were functioning normally and cell division was clearly observed. Daughter cells also exhibited fluorescence signal in the same wavelength region as parent cells.

In this approach, a highly focused high frequency ultrasound was used to deliver exogenous molecules into cell cytoplasm by disrupting the cell membrane similar to low frequency ultrasound and microbubble-based sonoporation^24^ and electroporation^11^. A highly focused high frequency ultrasonic transducer is capable of concentrating acoustic energy into a micrometer-sized region at desired locations without microbubbles, which are required for sonoporation using low frequency ultrasound. The diameter of the focused region of high frequency ultrasound corresponds to sub-cellular size (Supplementary Materials and Supplementary Figure 1). We hypothesize that the focused ultrasound energy perturbs the cell membrane and a passive diffusion, caused by the gradient of molecular concentration, induces intracellular delivery of molecules. The hypothesis is supported by influx of Ca^2+^, propidium iodide (PI), and 3 kDa dextran molecules. Live cell fluorescence images in Figs 2b and 5b, Supplementary Figure 4B, and Supplementary Video 3 confirm that perturbation of the cell membrane due to focused high frequency ultrasound is only limited to the target area because the changes of fluorescence intensity of PI starts at the targeted area and spreads out to the whole cell. The target area is determined by the focal area of the ultrasonic transducer, which is less than 10 μm in this study. Ca^2+^ influx patterns in Fig. 2d are different from those in Figs 4d and 5d. Rebound in FRET ratio plot in Fig. 2d after solid arrow indicates stronger perturbation or disturbance on cell plasma membrane after the application of an acoustic pulse. FRET ratio increases due to the coupling of delivered Ca^2+^ and FRET biosensors inside the targeted single-cell. According to this observation, two different mechanisms of the intracellular delivery of macromolecules can be drawn. The first case is to apply one strong acoustic pulse (PRF = 0, NP = 1, *V*_*pp*_ = strong, and *t*_*p*_ = strong) and the second case is to apply multiple and gentle acoustic pulses (PRF > 0 and NP > 1, *V*_*pp*_ = weak, and *t*_*p*_ = weak). The choice of pulse sequence may depend on the size and the amount of molecules to be delivered.

A diffusion-based intracellular delivery of exogenous molecules through a perturbed area on the cell membrane by high frequency ultrasound is vector-free and does not rely on foreign materials such as chemically modified molecules^38^ or lipids and polymers^7^,^8^. Compared to the low frequency ultrasound and microbubble-based sonoporation, a much simpler experimental protocol is required because the highly focused high frequency ultrasound approach does not rely on microbubbles. There is no need to understand complicated bubble dynamics under different insonation conditions and the interaction between microbubbles and cells. Spatially controlled delivery of exogenous molecules could also be achieved with high frequency ultrasound because of the controlled application of the acoustic pulse with sub-cellular resolution, which may not be possible in low frequency ultrasound and microbubble-based sonoporation due to the random attachments between microbubbles and cells.

The effects of acoustic pulses on targeted single-cells need to be estimated. Acoustic pressure field of ultrasonic transducers with the center frequency over 60 MHz cannot be directly measured with current technology. We explored the effects of acoustic pulses on targeted single-cells in three indirect ways. First, we developed 2D finite element acoustics model in PZFlex to simulate acoustic pressure field, generated by UT150 and UT215 (Supplementary Material and Supplementary Figure 2). According to the simulation results, the maximum pressure did not exceeded 5.6 MPa throughout experiments. When UT150 was excited by *Vpp* of 47 V, the strongest force, applied on targeted single-cells, was 440 μN (=pressure (5.6 MPa) × focal area (φ10 μm)). The value seems larger than the literature^24^,^39^, but it may be realistic. When sonoporation induced the influx of propidium iodide (PI) using microbubble and low frequency ultrasound, inertial cavitation was the main mechanism^24^. The equation for calculating acoustic radiation force on a microbubble^40^ is no longer valid anymore when cavitation occurs because the oscillation of the microbubble is in nonlinear regime and much larger than the initial radius of the microbubble. Shock wave is also followed by the cavitation. Therefore, acoustic forces can be much larger than nano-newton range for sonoporation or acoustic-transfection. Second, the upper limits of input parameters that induce cell death were investigated by a live cell imaging using FRET-based Ca^2+^ biosensors and PI (Figs 2 and 3, Supplementary Figures 4 and 5, and Supplementary Video 1) and the input parameters to generate much weaker acoustic pulses were used throughout experiments. Last, we performed short-term (6 hr) cell viability test (Supplementary Figure 9) and long-term (40 hr) cell tracking (Fig. 6 and Supplementary Figures 7 and 8) to examine the risk of impulsive sound pressure, which may be the case for this study, of causing physical damage to targeted single-cells.

Dextran was chosen as an example of macromolecules to be delivered into the cytoplasm of cells because it is an inert and hydrophilic molecule, which is not toxic inside cells^41^. It was reported that the stokes’ radii of Alexa is approximately 0.6 nm and molecular weight is 550 Da^42^. The maximum diameter of a pore needed, allowing propidium iodide (PI) to pass, is approximately 1.5 nm^37^. 3 kDa dextran has a hydrodynamic radius of about 2.7 nm, which is similar in size to siRNA^43^,^44^. PI transport across the cell membrane was achieved with relatively short acoustic pulses compared to 3 kDa dextran. 3 kDa dextran is small enough to passively be transported through the nuclear envelope after it is delivered into cell cytosol^41^,^45^. These results suggest robust intracellular delivery mechanisms of extracellular cargoes when cells are subjected to focused ultrasound perturbation. The initial findings seem to suggest that the delivery of exogenous molecules may be induced by forming transient holes in the lipid bilayer of the cell membrane, but this observation needs to be further confirmed.

Optimization of input parameters of acoustic-transfection system will be performed as a future work to investigate the effects of heterogeneity within a same cell type and between different kinds of cell types to the intracellular delivery of macromolecules using acoustic-transfection. We will develop next generation acoustic-transfection system with high-throughput. By combining these capabilities including single-cell level targeting, vector-free, low cytotoxicity, and high-throughput, the acoustic-transfection technique may provide a new method complementary to other transfection methods.

Regenerative medicine and gene editing can be benefited from the single-cell level targeting of acoustic-transfection technique. Only specifically targeted cells need to be monitored after delivering transcription factors or CRISPR/Cas9 system instead of searching transfected cells out of thousands of cells. Another advantage of single-cell level transfection over bulk transfection is that two neighboring single cells can express different cargo (or biosensor) proteins and allow simultaneous monitoring of different molecular events in these neighboring cells. For *in vivo* application, catheter type ultrasonic transducer^46^,^47^ can manipulate cells deep inside organ. An array type ultrasonic transducer can increase throughput. For example, a 10 by10 or 20 by20 array has 100 or 400 times higher throughput than the current single element transducer. After suspended cells are trapped inside wells in a microfluidic chip, acoustic-transfection may be used to deliver target molecules into targeted cells.

## Materials and Methods

### High frequency ultrasonic transducer

Ultrasonic transducer 150 (UT150) and 215 (UT215) were designed and fabricated with conventional transducer fabrication procedures^35^. Briefly, lithium niobate (LiNbO_3_, PZT in Supplementary Figure 1) was lapped down to 10 μm. A conductive silver epoxy was cast on the lapped PZT to form a backing layer (BL in Supplementary Figure 1). The stack of PZT and BL was turned down to a cylindrical shape with 1 mm diameter and 1 mm height. Back side of BL was connected to a wire and the whole structure was placed into a housing with 1.65 mm diameter using insulation epoxy (IE in Supplementary Figure 1). IE provided the structural integrity between the stack of PZT and BL and the housing as well as electrical insulation. The open surface of PZT was press-focused using a 2 mm diameter stainless steel ball to make a focus at 1 mm away from the PZT surface. An aperture of 1.0 mm and a focal distance (FD, Supplementary Figure 1) of approximately 1.0 mm resulted in *f*_*number*_ of approximately 1.0. The pulse-echo time response and its spectrum for UT150 and UT215 were measured and the center frequencies of UT150 and UT215 were estimated to be 150 MHz and 215 MHz, respectively, as shown in Supplementary Figure 1.

### Acoustic-transfection system

We adapted the high frequency ultrasound microbeam stimulation (HFUMS) system^48^ by integrating UT150 and UT215 with new fluorescence microscopes as shown in Fig. 1 for improved single-cell level targeting and simultaneous live cell imaging using fluorescence resonance energy transfer (FRET)-based biosensors and propidium iodide (PI). Acoustic-transfection system 1 (ATS1) consisted of a Nikon microscope (Eclipse Ti-U, Melville, NY) and UT150. Acoustic-transfection system 2 (ATS2) consisted of Leica microscope (Leica DMI 4000B, Germany) and UT215. ATS1 was used for FRET-based Ca^2+^ and PI imaging and ATS2 was for the intracellular delivery of 3 kDa dextran and cell viability test. In addition to ultrasonic transducers and microscopes, ATS1 and 2 also included a 3D translation/rotation stage, a pulser/receiver and an oscilloscope to place the focus of an ultrasonic transducer at a targeted single-cell, and a function generator and a power amplifier to generate electric pulse (EP in Fig. 1a) to trigger an ultrasonic transducer as shown in Fig. 1.

### Targeting a single-cell

An ultrasonic transducer (UT150 or UT215) was attached to a 3D translation/rotation stage to control the transducer position of the ATS as shown in Fig. 1a. The ultrasonic transducer was located within the microscope’s field of view to allow monitoring and recording the movement of the transducer using the microscope. The ultrasonic transducer was connected to a pulser/receiver and an oscilloscope. Once a petridish, filled with only water, was placed on the microscope stage, the tip of the ultrasonic transducer was immersed into water to locate the transducer’s focus on the surface of the petridish (Fig. 1b). When the maximum amplitude of echo time response was observed on an oscilloscope (Fig. 1a and Supplementary Figure 1), the transducer remained stationary and the location of the transducer was recorded so that the ultrasonic transducer was able to return to the position later during the experiments.

### Cell culture, delivery molecules, and FRET-based Ca^2+^ biosensors

Human cervical carcinoma (HeLa, American Type Culture Collection, Manassa, VA) cells were incubated at 37 °C and 5% CO_2_ in EMEM supplemented with 10% FBS. Cells were seeded in a 35 mm petridish to make a monolayer of cells 36 hours before performing experiments. Propidium iodide (Life Technologies, Eugene, OR) and 3 kDa dextran labeled with Alexa 488 (Life Technologies, Eugene, OR) for the intracellular delivery of macromolecules were purchased. FRET-based Ca^2+^ biosensor has been developed by our group and used (Supplementary Figure 3)^6^,^49^. FuGene HD Transfection Reagent was used to transfect FRET-based Ca^2+^ biosensors into HeLa cells 24 hours before the experiments according to manufacturer’s manual. A LIVE/DEAD Cell Imaging kit (Life Technologies, Eugene, OR) was obtained for a short-term cell viability test.

### Live cell imaging and image analysis

Simultaneous live cell imaging using FRET-based Ca^2+^ biosensors and propidium iodide (PI) was performed. We prepared 100 μM of PI by mixing PI molecules with HBSS with Ca^2+^ (Life Technologies, Eugene, OR). We added 1.6 ml of PI solution into the previously prepared petridish with a monolayer of HeLa cells, transfected with FRET-based Ca^2+^ biosensors. The petridish was placed on the microscope stage. Acoustic-transfection system 1 (ATS1) with a charge-coupled device (CCD) camera was used. Images and plots of FRET-YFP/CFP ratio and PI intensity were generated using ImageJ software^50^ and Matlab (Mathworks). Region of interest 1 (ROI 01) corresponded to the location, where an acoustic pulse was applied, with the diameter of 10 μm. ROI 02 and 03 were selected to make concentric circles with ROI 01 with the diameters of 15 μm and 20 μm.

### Intracellular delivery of 3 kDa dextran and 40 hours of cell tracking

ATS 2 was used for the intracellular delivery of 3 kDa dextran. Two milligrams of 3 kDa dextran labeled with Alexa 488 were diluted with 13.3 milliliters of HBSS with Ca^2+^ to make 50 μM dextran solutions. The prepared 3 kDa dextran solution was added into the petridish with a monolayer of HeLa cells without Ca^2+^ biosensors this time after the cells were washed three times with HBSS Ca^2+^. We targeted a single-cell using previously described method above. We fixed the peak-to-peak voltage (*V*_*pp*_) at 22 V and two different pulse durations (*t*_*p*_) of 16 μs and 23 μs were used (PRF = 0 and NP = 1 in Fig. 1a). Ten cells were treated for each case (n = 10). A bright-field image was acquired right before an acoustic pulse was applied. The cells were incubated at 37 °C and 5% CO_2_ in the 3 kDa dextran solution for 5 minutes after treatment to ensure resealing of the membrane holes and then washed three times with HBSS with Ca^2+^. The washed cells were incubated again at 37 °C and 5% CO_2_ in culture medium for 25 minutes. Approximately 30 minutes after the treatment, bright-field and fluorescence images were captured and saved. Treated cells were incubated in culture medium for 39 more hours and imaged to track cell conditions. Images were captured by a high sensitivity and high resolution EM-CCD camera and saved still shot capture of bright field and fluorescence images for off-line analysis using ImageJ software^50^.

### Short-term (6 hr) cell viability test

Short-term (6 hr) cell viability was performed before the intracellular delivery of 3 kDa dextran to test the cytotoxicity of applied acoustic pulses. The input parameters of an acoustic pulse were same as that for 3 kDa dextran experiments (*V*_*pp*_ = 22 V, *t*_*p*_ = 16 μs and 23 μs, PRF = 0, and NP = 1). Cell viability test was performed with a different batch of cells. Cells in the control group (CG) were not treated with an acoustic pulse (*t*_*p*_ = 0 μs) and cells in the treatment groups 1 (TR1) and 2 (TR2) were exposed to acoustic pulses with *t*_*p*_ of 16 μs and 23 μs, respectively. CG and TR 1 and 2 followed the same experimental protocol. The number of cells in each group was 18. Bright-field images of target cells were taken as a reference (t = 0 hr). After the treatment, cells were incubated at 37 °C and 5% CO_2_ in culture medium for 6 hours. After growth media was completely washed out three times with HBSS with Ca^2+^, cells were stained with a LIVE/DEAD Cell Imaging kit, according to the manufacturer’s instructions and imaged on the microscope to determine whether cells were alive (green) or dead (red). The number of cells with green fluorescence after 15 minutes was counted and cell viability was calculated as the ratio between the numbers of live cells to the total number of cells in percentage.

## Additional Information

**How to cite this article**: Yoon, S. *et al*. Direct and sustained intracellular delivery of exogenous molecules using acoustic-transfection with high frequency ultrasound. *Sci. Rep.* **6**, 20477; doi: 10.1038/srep20477 (2016).

## Supplementary Material

Supplementary Video 1

Supplementary Video 2

Supplementary Video 3

Supplementary Information
