# Supplementary material for: Direct and sustained intracellular delivery of exogenous molecules using acoustic-transfection with high frequency ultrasound
Source: Sci Rep. 2016 Feb 4;6:20477. doi: 10.1038/srep20477 (PMC4740885; doi:10.1038/srep20477)
Supplement: Supplementary Information [file srep20477-s4.pdf]

Direct and sustained intracellular delivery of exogenous molecules using acoustic-transfection with high frequency ultrasound

Sangpil Yoon[1], Min Gon Kim[1], Chi Tat Chiu[1], Jae Youn Hwang[2], Hyung Ham Kim[1], Yingxiao Wang[3], K. Kirk Shung[1,*]

[1]Department of Biomedical Engineering, University of Southern California, Los Angeles, California 90089, USA

[2]Department of Information and Communication Engineering, Daegu Gyeongbuk Institue of Science & Technology, Daegu, Korea

[3]Department of Bioengineering & Institute of Engineering in Medicine, University of California, San Diego, La Jolla, California 92093, USA

[*]Corresponding Author: K. Kirk Shung, Ph.D., 1042 Downey Way, DRB-136, Los Angeles CA, 90089; kkshung@usc.edu; +1-213-821-2653

Supplementary Materials

Simulation of acoustic pressure field

A finite element model was developed using commercial virtual prototyping tool PZFlex (Weidlinger Associates, Inc., USA.) to estimate the acoustic pressure field of the ultrasonic transducers 150 (UT150) and 215 (UT215) of the acoustic-transfection systems under ideal conditions. The transducer model was generated based on the actual design parameters and immersed in a homogeneous loading medium (i.e. water in this model). The grid size was set to be 1/15 of the model wavelength. The attenuation coefficient of water for the simulation was assumed to be 0.002 dB/cm/MHz. Different electrical loading conditions were then applied across the transducer electrodes for comparisons. Specifically, the peak-to-peak input voltages (V_{pp}) were 22 V and 47 V in accordance with the actual experimental values. Pulse durations (t_p) were 6.7, 13, 20, 33, 73, and 147 nanoseconds for UT150 and 4.6, 9.3, 14, 23, 51, and 102 nanoseconds for UT 215, which corresponded to 1, 2, 3, 5, 11, and 22 cycles. The propagation of pressure into water was computed and the maximum / minimum acoustic pressure fields were determined.

2D plots in Supplementary Figures 2B and 2C show simulated acoustic pressure fields, generated by UT215 with V_{pp} of 22 V and t_p of 102 ns and UT150 with V_{pp} of 47 V and t_p of 147 ns, respectively. Axial and lateral pressure profiles of two cases show the foci of UT150 and UT215 clearly. Sound waves propagate along +x direction in 2D plots in Supplementary Figures 2B and 2C. Simulation results show that the maximum values of the simulated pressure reach saturation point (dash-dotted line in Supplementary Figure 2A) with t_p of approximately 30 nanoseconds. The maximum pressure does not exceed 5.6 MPa. The estimated diameters at the

focus by simulation and theory are 9 μm and 7 μm for UT215 and 12 μm and 10 μm for UT150, respectively.

Supplementary Figure Captions

Supplementary Figure 1. An ultrasonic transducer and an acoustic pulse. (A) 3D view and (B) schematic view of detailed structure of a high frequency ultrasonic transducer. We fabricated ultrasonic transducers using lithium niobate (LiNbO3, PZT) and the diameter of housing was 1.65 mm. Backing layer (BL) attenuates unwanted signals, generated by PZT. Electrode and a wire are for electrical connection through SMA connector. Insulation epoxy (IE) fixes the stack of PZT and BL and provides insulation between positive and negative electrical connections. Aperture is 1.0 mm and focal distance (FD) is 1.0 mm. Parylene coating (PC) is a protective layer from corrosion and water. Echo time response (dashed lines) and it spectrum (solid lines) of (C) ultrasonic transducer 150 (UT150, center frequency= 150 MHz) and (D) ultrasonic transducer 215 (UT215, center frequency= 215 MHz) were measured with 0 dB gain and 20 dB gain, respectively. (E) Circular permanent marks are generated on a plastic petridish by acoustic pulses from UT150 with t_p = 60 μs, PRF = 0, and NP = 1 and the diameter of circular marks depends on V_{pp} from 40 V to 70 V. (F) A circular mark is smaller than a HeLa cell. The scale bar represents 20 μm.

Supplementary Figure 2. Simulated acoustic pressure field of the ultrasonic transducers 150 (UT150) and 215 (UT215). (A) The maximum values of simulated pressure at the focus of UT150 and UT215 are plotted with respect to pulse durations (t_p). The maximum values reach saturation values when UT150 and UT215 are excited by t_p of approximately 30 ns (dash-dotted line). Two representative examples of 2D acoustic pressure field are simulated after (B) UT215 and (C) UT150 are excited with the input parameters of V_{pp}= 22 V and t_p= 102 ns and V_{pp}= 47 V and t_p= 147 ns, respectively. Sound waves propagates +x direction and 2D plots start from

aperture. Axial and lateral pressure profiles show the foci of UT215 and UT150 near 1 mm away from aperture.

Supplementary Figure 3. A schematic drawing of the activation of FRET-based Ca^{2+} biosensor after the introduction of $Ca^{2+.}$

Supplementary Figure 4. Upper limit of input parameters not to induce cell death with V_{pp} of 47 V. (A) Color images and (D) the time courses of the FRET-YFP/CFP ratio of Ca^{2+} biosensor at region of interest (ROI) 01, 02, and 03 show rapid increase of the intracellular Ca^{2+} concentration right after the application of an acoustic pulse with input parameters of t_p= 10 μs, PRF = 0, and NP = 1. A rebound of FRET-YFP/CFP ratio after the solid arrow at (D) indicates that Ca^{2+} influx continues. The color scale bar on the left represents the range of FRET ratio from black (low Ca^{2+} concentration) to red (high Ca^{2+} concentration). (B) Color images and (E) the time courses of PI intensity at the same ROIs represent that the PI molecules spreads throughout the whole cell after a strong influx of PI molecules through ROI 01. The color scale bar indicates the range of PI intensity with blue (low) and red (high) levels of PI concentration. The image in (C) show DIC image. The scale bar represents 10 μm. The error bars represent plus and minus one standard deviation. Arrow head in (D) indicates the application of acoustic pulse.

Supplementary Figure 5. Cell death observation by strong acoustic pulse with V_{pp} = 47V. (A) Color images and (C) the time courses of the FRET-YFP/CFP ratio of Ca^{2+} biosensor at region of interest (ROI) 01, 02, and 03 represent cell death. White solid arrows indicate cell blebbing. The input parameter of an acoustic pulse is (t_p= 12 μs, PRF = 0, and NP = 1). The color scale bar on the left at (A) represents the range of emission ratio with black and red colors, indicating low and high levels of Ca^{2+} concentration, respectively. (B) Color images and (D) the

time courses of PI intensity at the same ROIs, obtained by a simultaneous live cell imaging, show extremely strong influx of PI molecules and continuous increase in PI intensity. This is the first evidence of the cell death and irreversible cell membrane disruption. The color scale bar indicates the range of PI intensity with blue (low) and red (high) levels of PI concentration. Color images of (E) FRET-YFP, (F) CFP, and (G) the time courses of two channels represent rapid decrease in FRET-YFP and CFP. FRET-YFP/CFP ratio plot in (C) can be misinterpreted that the targeted single-cell is alive because the FRET ratio decreases as already seen in Figures 2, 4, and 5. However, the decrease in FRET ratio in (C) comes from CFP decrease (third panel in (F)), followed by FRET-YFP decrease, due to an irreversible cell membrane disruption. The scale bar represents 10 μm. The error bars represent plus and minus one standard deviation. Arrow head in (C) indicates the application of acoustic pulse.

Supplementary Figure 6. Cell death observation by strong acoustic pulse with V_{pp} = 22V. Color images of (A) FRET-YFP, (B) CFP, and (C) the time courses of two channels have different behavior compared to Supplementary Figure 5. CFP intensity leakage is not observed at (B), but a continuous increase in FRET-YFP represents unceasing influx of Ca^{2+}. The error bars represent plus and minus one standard deviation. The scale bar represents 10 μm.

Supplementary Figure 7. Intracellular delivery of 3 kDa dextran by an acoustic pulse with V_{pp} = 22V and t_p = 16 μs. 90% of targeted single-cells are successfully transfected with 3 kDa dextran labeled with Alexa 488 using acoustic-transfection system 2 (ATS2, n=10). One case is in Figure 6A and eight cases are shown here. The first and the second rows are fluorescence and bright-field images of targeted single-cells 30 minutes and 40 hours after the treatment. Daughter cells emit fluorescence signal in the same wavelength region as parent cells after 40 hours and they are functioning normally.

Supplementary Figure 8. Intracellular delivery of 3 kDa dextran by an acoustic pulse with V_{pp} = 22V and t_p = 23 µs. 80% of targeted single-cells are successfully transfected with 3 kDa dextran labeled with Alexa 488 using acoustic-transfection system 2 (ATS2, n=10). One case is in Figure 6B and seven cases are shown here. The first and the second rows are fluorescence and bright field images of targeted single-cells 30 minutes and 40 hours after the treatment. Daughter cells emit fluorescence signal in the same wavelength region as parent cells after 40 hours and they are functioning normally.

Supplementary Figure 9. Short-term (6 hr) cell viability test. The control group (CG, n=18, t_p = 0 µs) and treatment groups 1 (TR1; n=18; V_{pp}= 22 V, t_p= 16 µs) and 2 (TR2; n=18; V_{pp}= 22 V, t_p= 23 µs) are used for short-term (6 hr) cell viability test. One representative image of a targeted single-cell, indicated as arrows, from (A) TR1 and (B) TR2 are presented. Images in the left column show target cells right after the treatment (t= 0 hr) and images in the middle and right columns present bright-field and green fluorescence images of targeted single-cells 6 hours after the treatment. (C) Cell viability of all groups is estimated as 100%. (Scale bar, 20 µm).

Supplementary Video Captions

Supplementary Video 1. FRET-YFP/CFP ratio video shows cell death after the application of a strong acoustic pulse with V_{pp}= 47 V and t_p= 12 µs. A HeLa cell, transfected with FRET-based Ca^{2+} biosensor, shows strong increase in FRET-YFP/CFP ratio and cell blebbing around the cell boundary is also observed. This is the same cell in Supplementary Figure 4. Total duration: 235 sec.

Supplementary Video 2. Five repeated applications of an acoustic pulse with V_{pp}= 47 V and t_p= 2 µs induce recurring increase and decrease in FRET-YFP/CFP ratio. A HeLa cell, transfected with FRET-based Ca^{2+} biosensor, is used and FRET-YFP/CFP ratio returns to normal level, which indicates the reversible perturbation of cell plasma membrane. This is the same cell in Figure 5. Total duration: 1312 sec.

Supplementary Video 3. Each acoustic pulse with V_{pp}= 47 V and t_p= 2 µs induces small amount of intracellular delivery of propidium iodide molecules. This video was acquired from the same imaging session with the video in Supplementary Video 2 using RFP channel. After five applications of an acoustic pulse, accumulated PI molecules show significant fluorescence signal. Total duration: 1312 sec.

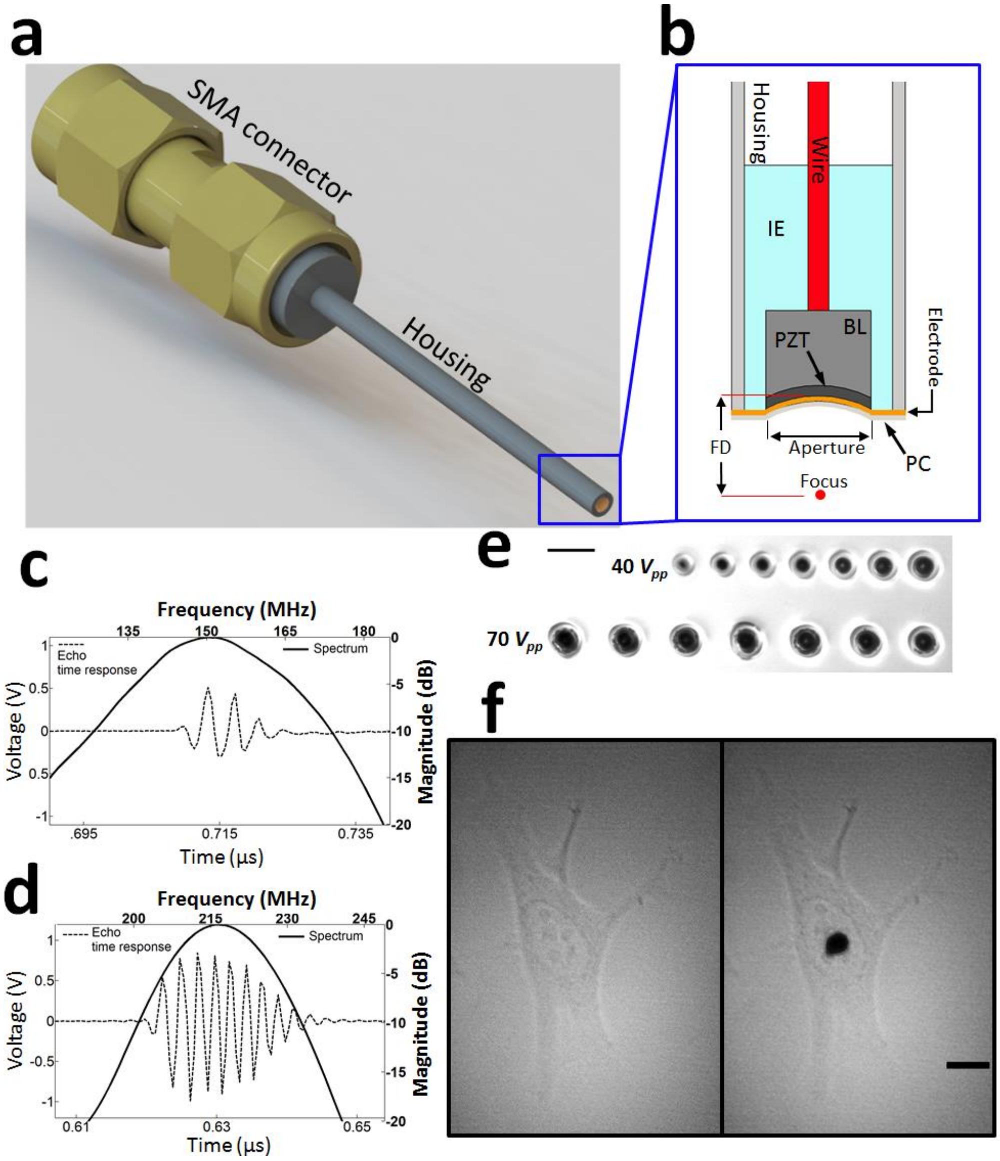

Supplementary Figure 1.

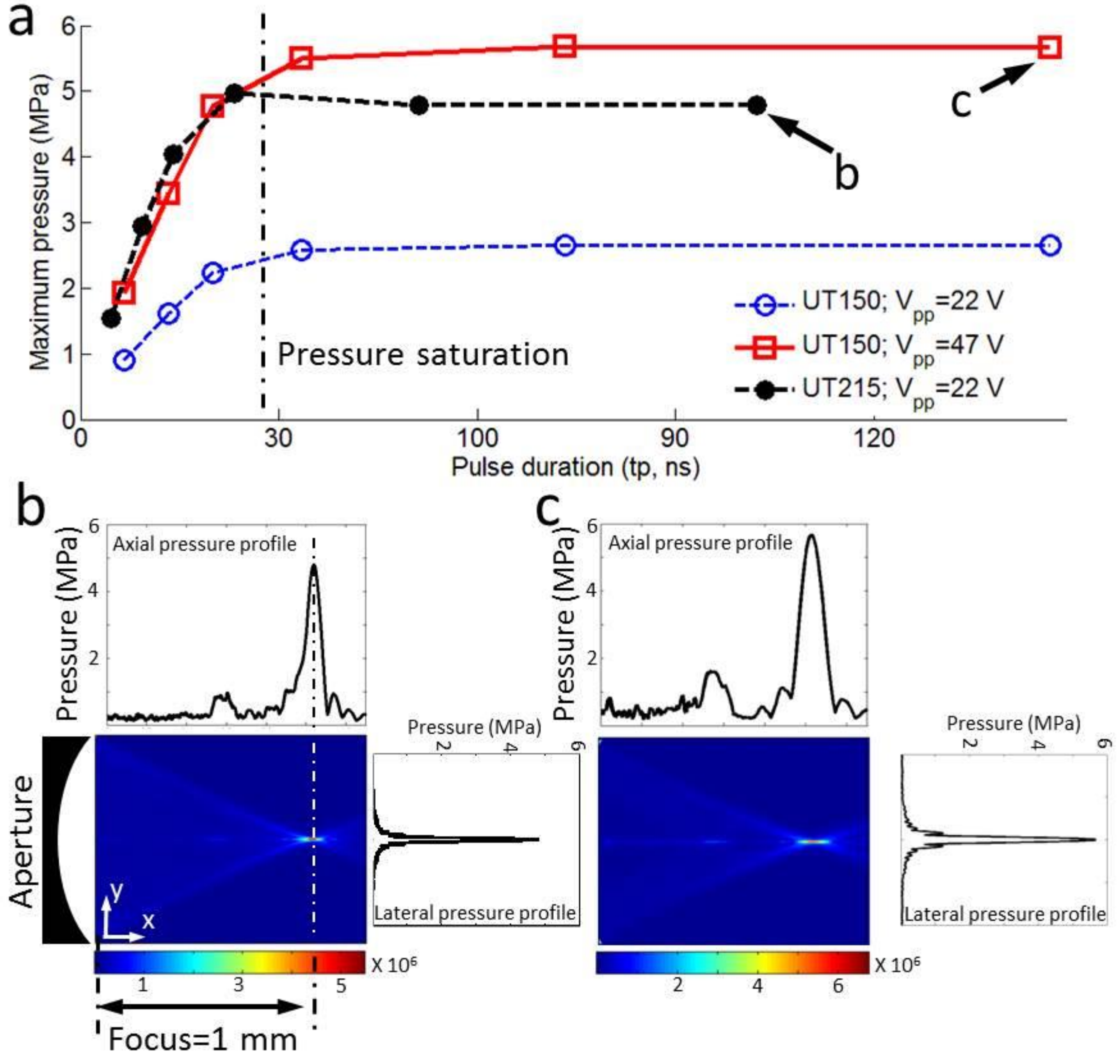

Supplementary Figure 2.

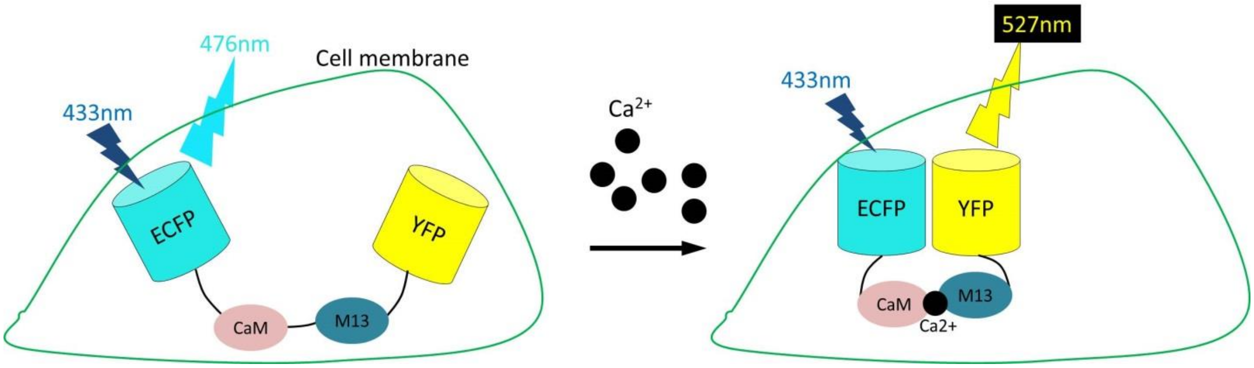

Supplementary Figure 3.

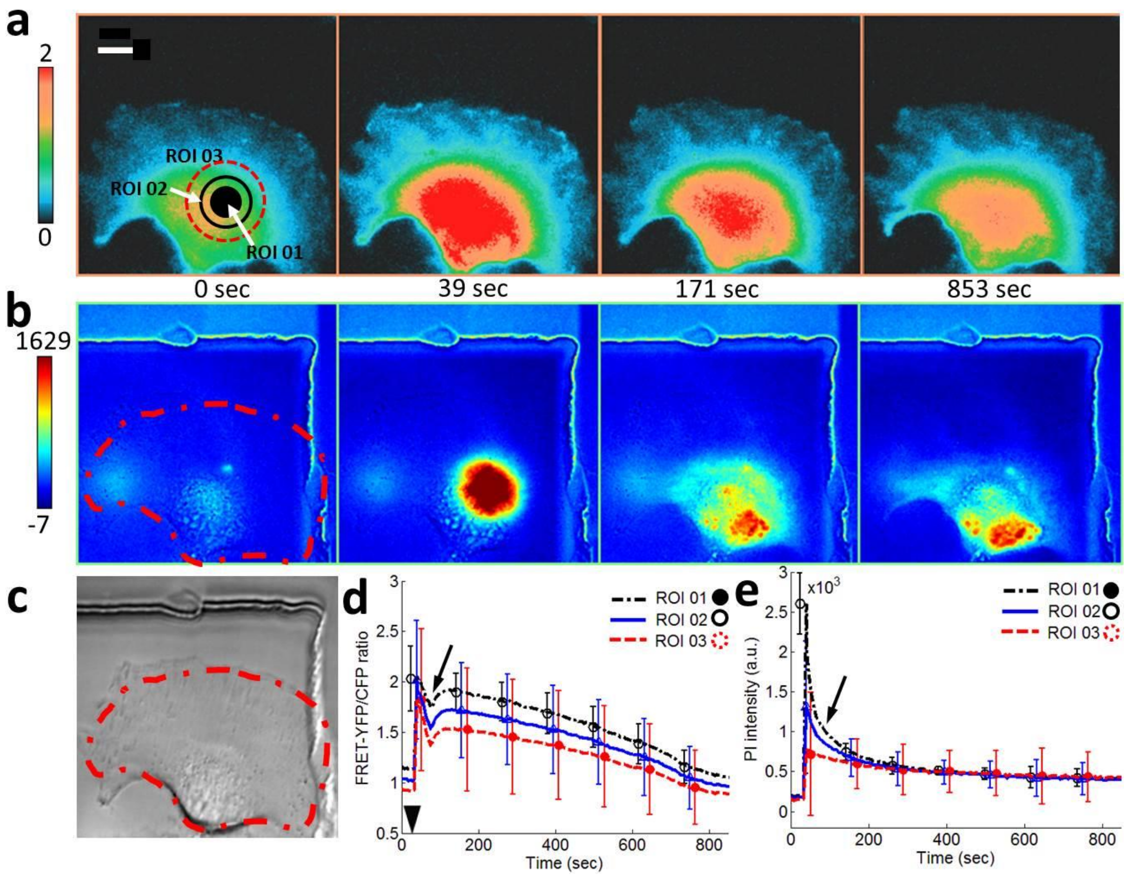

Supplementary Figure 4.

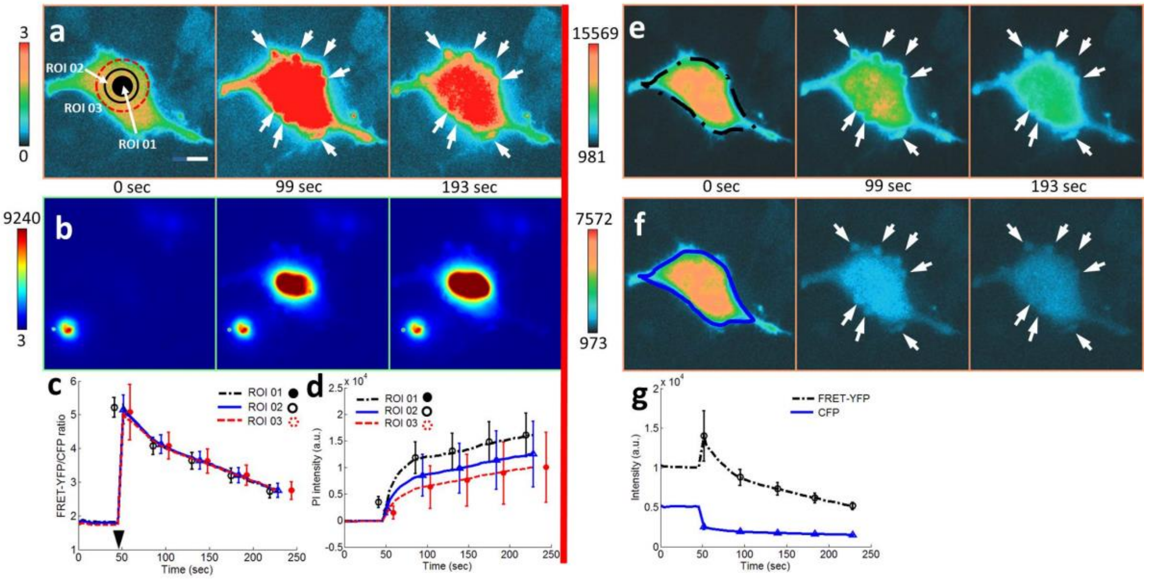

Supplementary Figure 5.

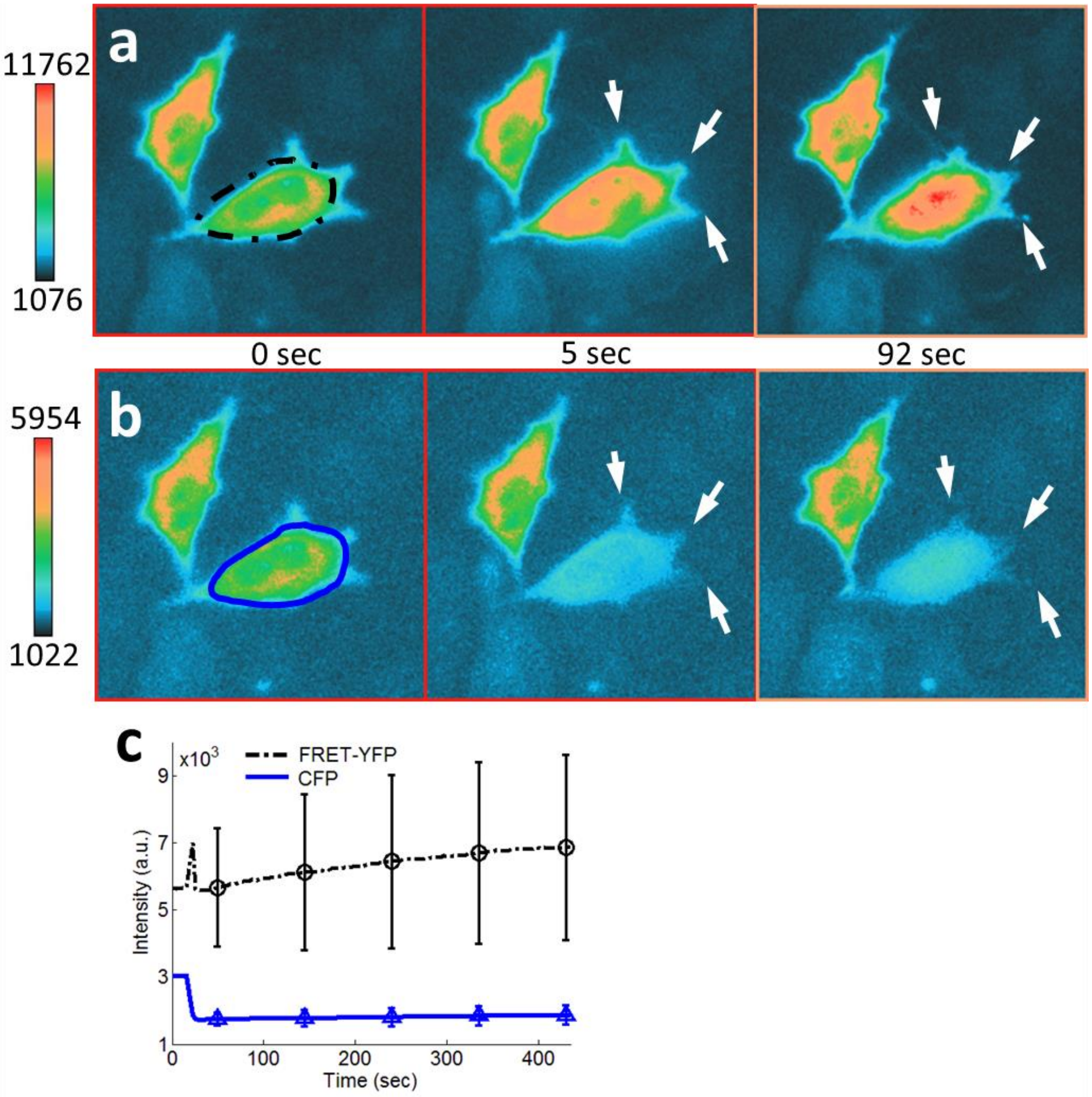

Supplementary Figure 6.

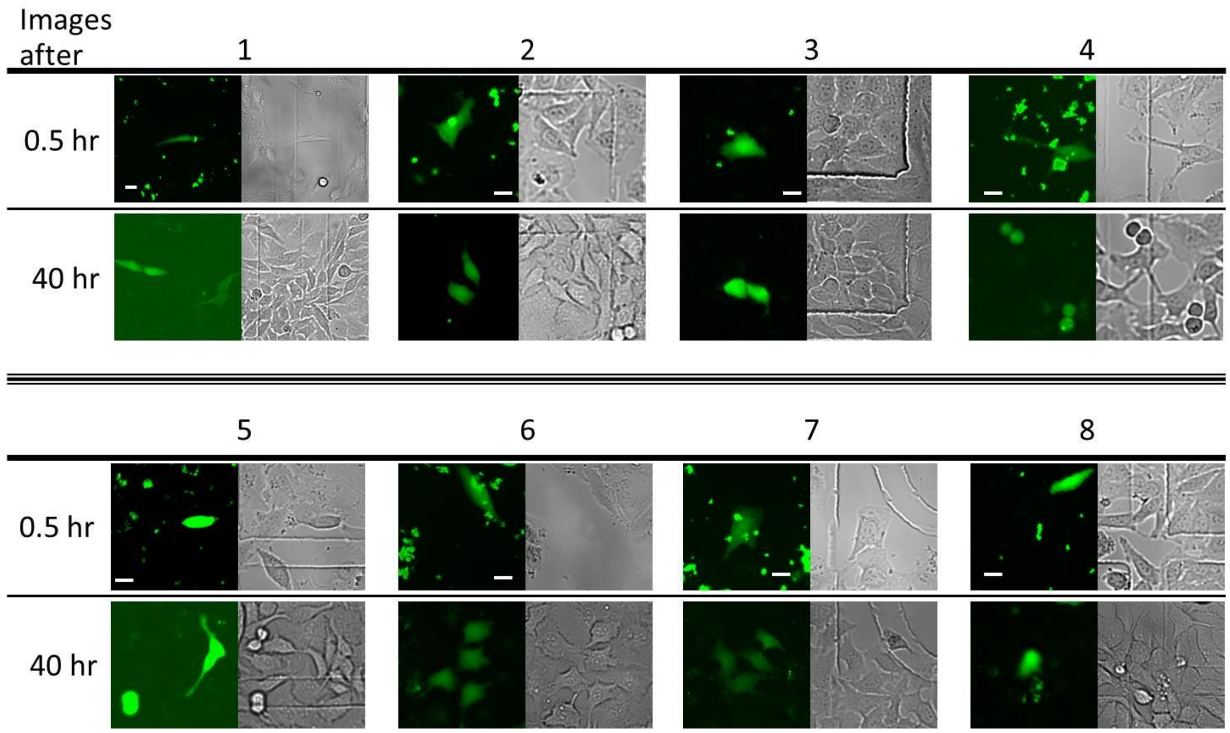

Supplementary Figure 7.

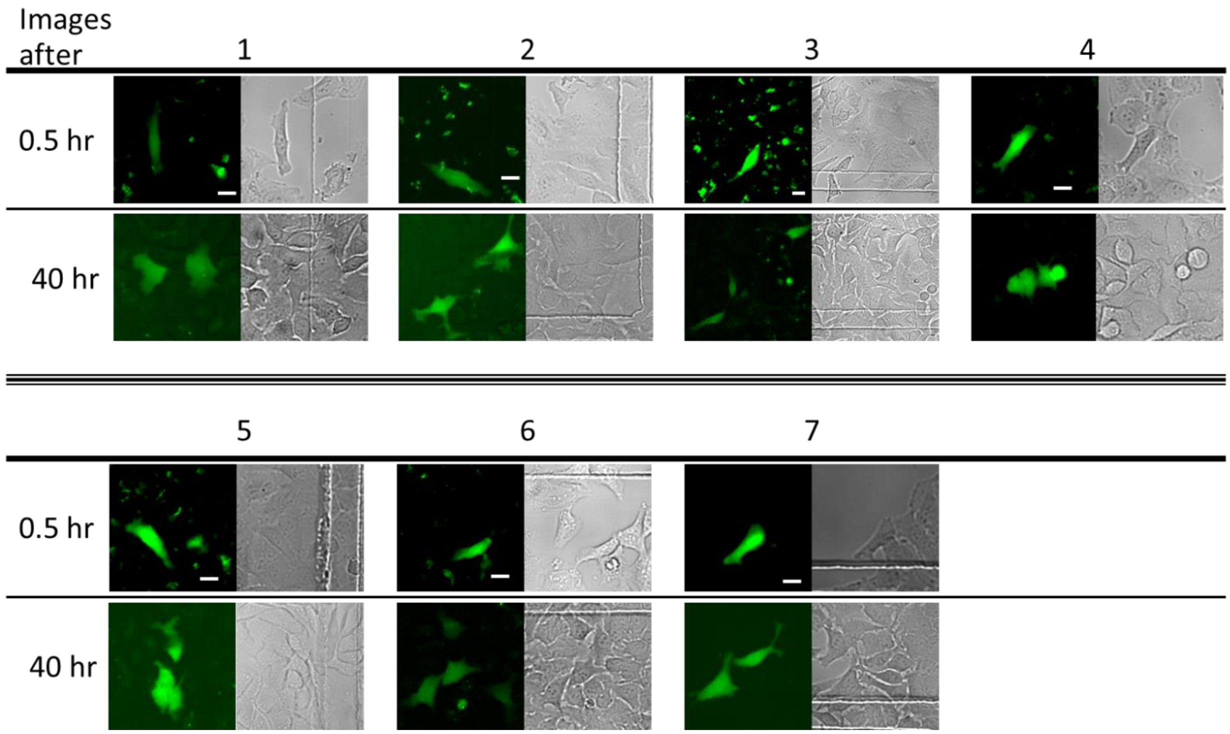

Supplementary Figure 8.

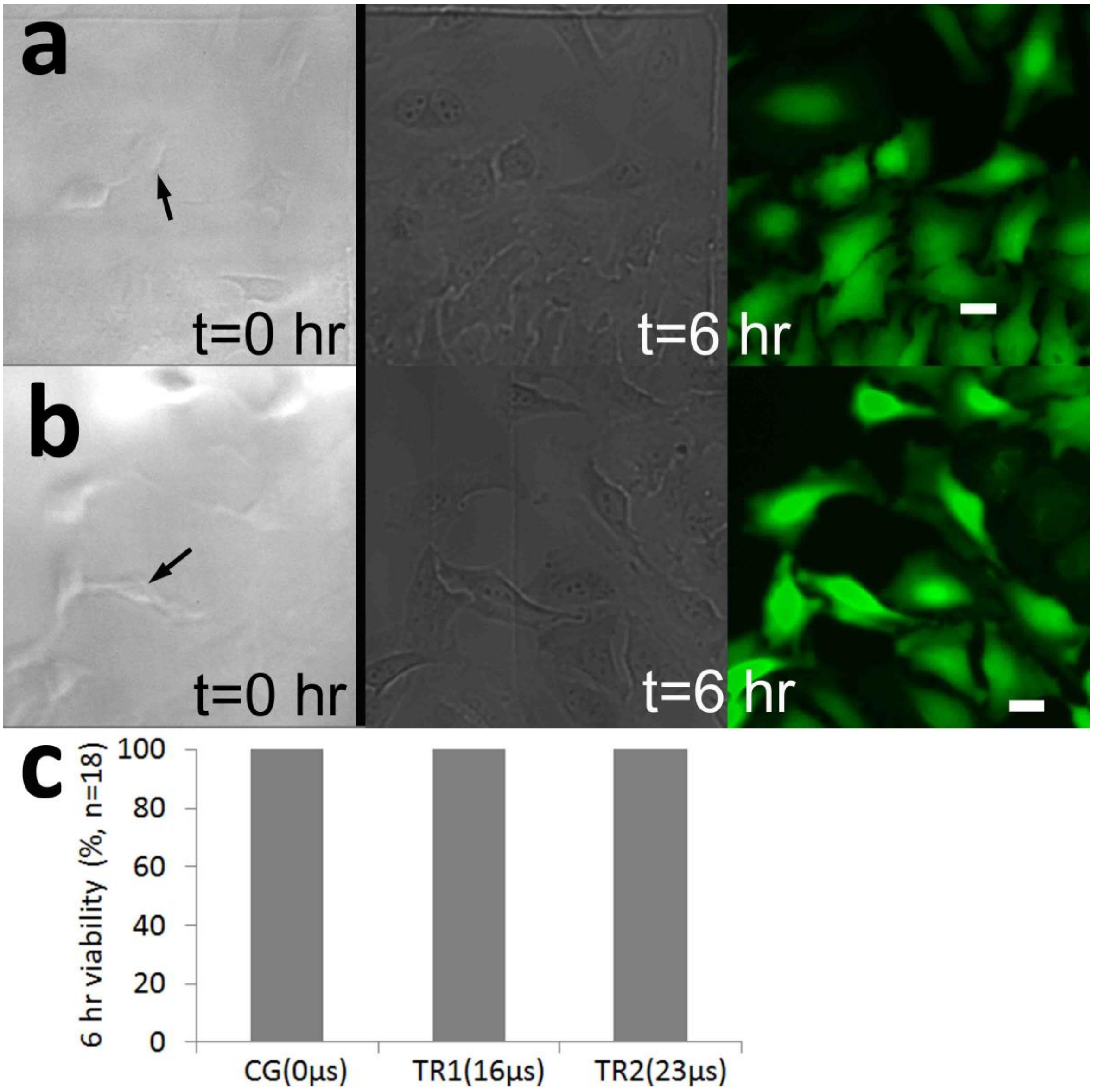

Supplementary Figure 9.
